# Characteristics of Pompe disease in China: a report from the Pompe registry

**DOI:** 10.1186/s13023-019-1054-0

**Published:** 2019-04-03

**Authors:** Yuying Zhao, Zhaoxia Wang, Jiahong Lu, Xuefan Gu, Yonglan Huang, Zhengqing Qiu, Yanping Wei, Chuanzhu Yan

**Affiliations:** 1 Shandong University Qilu Hospital, No.107 Wen Hua Xi Road, Jinan, Shandong Province China; 2 Beijing University 1st hospital, No. 8 Xi Shi Ku Street, Beijing, China; 3 Fudan University Huashan hospital, No. 12 Middle Wulumuqi Road, Shanghai, China; 4 Xinhua Hospital, School of Medicine, Shanghai Jiao Tong University, No. 1665 Kong Jiang Road, Shanghai, China; 5 Guangzhou Women & Children’s Medical Center, Xing Nan Avenue, Guangzhou, China; 6 Peking Union Medical College Hospital, No. 9 Dongdan Santiao, Beijing, China

**Keywords:** Pompe disease, Pompe registry, China, Late onset Pompe disease

## Abstract

**Background:**

Pompe disease is a rare, progressive, autosomal recessive lysosomal storage disorder caused by mutations in the acid α-glucosidase gene. This is the first report of Chinese patients from the global Pompe Registry. Chinese patients enrolled in the Registry (ClinicalTrials.gov, NCT00231400) between Jan 2013 and 2 Sep 2016 with late onset Pompe disease (LOPD; presentation after 12 months of age or presentation at ≤12 months without cardiomyopathy) were included. Data analyses were descriptive.

**Results:**

Of the 59 Chinese patients included, 86.4% had never received enzyme replacement therapy (ERT). The age at symptom onset and diagnosis was 14.9 (12.35) and 22.1 (10.08) years, which is younger than previous reports of LOPD patients from the rest of the world (28.4 [18.86] and 34.9 [20.03], respectively). The most common diagnosis methods were enzyme assay (79.7%) and/or DNA analysis (61.0%). Of the 36 patients diagnosed using DNA analysis, 31 had standardized variant data and among these patients the most common mutations were c.2238G > C (*n* = 18, 58.1%) and c.2662G > T (*n* = 5, 16.1%). Chinese LOPD patients appeared to have worse lung function versus patients from the rest of the world, indicated by lower forced vital capacity (37.2 [14.00]% vs. 63.5 [26.71]%) and maximal expiratory and inspiratory pressure (27.9 [13.54] vs. 51.0 [38.66] cm H_2_O, and 29.4 [12.04] vs. 70.5 [52.78] cm H_2_O).

**Conclusions:**

Compared with patients from the rest of the world, Chinese patients with LOPD appeared to have younger age at symptom onset and diagnosis, lower lung function, and the majority had not received ERT. The most common mutations were c.2238G > C and c.2662G > T.

**Electronic supplementary material:**

The online version of this article (10.1186/s13023-019-1054-0) contains supplementary material, which is available to authorized users.

## Background

Pompe disease (glycogen storage disease II, 232300) is a rare, progressive, autosomal recessive lysosomal storage disorder which is often fatal [1]. Mutations in the gene encoding for the lysosomal hydrolase acid α-glucosidase (GAA; EC 3.2.1.20) result in a deficiency of GAA and lysosomal accumulation of glycogen, particularly in cardiac and skeletal muscle and the nervous system, leading to cellular dysfunction and muscle damage [2, 3]. The incidence of Pompe disease is estimated to be 1 in 40,000 live births [4, 5]. However, neonatal screening programs conducted in Austria and Taiwan reported a much higher incidence of 1 in 8684 and 1 in 17,000, respectively [6, 7]. Before the development of enzyme replacement therapy (ERT) with recombinant human GAA (rhGAA), treatment of Pompe disease was limited to palliative care. ERT has been shown to increase GAA activity in muscle, heart and liver, and leads to glycogen clearance in cardiac muscle and the liver [8]. Currently, the only approved ERT for Pompe disease is Chinese hamster ovary-derived recombinant GAA (alglucosidase alfa, Myozyme®/Lumizyme®, Genzyme, Cambridge, MA, USA).

Pompe disease encompasses a spectrum of clinical manifestations which vary by age of onset, organ involvement and degree of myopathy [1, 8]. In general, Pompe disease is classified either as infantile onset (IOPD), often defined as onset of disease at age ≤ 12 months with cardiomyopathy (also known as ‘classic Pompe disease’), or late onset (LOPD), with presentation after > 12 months of age or presentation at ≤12 months without cardiomyopathy (also referred to as ‘non-classic’ Pompe disease). However, a variety of definitions are used and patients with onset of disease at ≤12 months without cardiomyopathy can also be categorized as atypical IOPD [9]. IOPD is usually characterized by a total loss of GAA enzyme activity and the most common symptoms include hypotonia, progressive weakness, macroglossia, hepatomegaly and hypertrophic cardiomyopathy. Life expectancy for IOPD is around 1 year, and death is usually due to cardio–respiratory failure [8, 10, 11]. In contrast, LOPD is generally associated with a wider range of age of onset and clinical symptoms, and the level of reduction of GAA enzyme activity shows inter-patient heterogeneity [8]. Common symptoms of LOPD include progressive limb girdle weakness and respiratory insufficiency, without cardiomyopathy [12].

Given the low prevalence of Pompe disease, there are relatively few data describing the phenotypes, disease course, and effectiveness of management strategies and treatments for this disorder among individuals from the Asia Pacific region and China. Almost all published data from Chinese people with Pompe disease have been case studies or case series and have included relatively small numbers (< 30) of patients [13–16]. While these previous studies are extremely valuable, they were predominantly focused on molecular and genetic investigations and there remains an unmet need for data describing phenotype and disease course. Given this situation, more comprehensive data from a larger population of Chinese people with Pompe disease would be of great clinical utility.

The Pompe Registry (herein referred to as the Registry) was initiated in September 2004 and represents the largest collection of data for patients with Pompe disease in the world. The Registry was designed to develop a better understanding of the natural history and outcomes of patients with Pompe disease. China joined the Registry in 2012, with the first patient enrolled in 2013. This report describes the key demographic and clinical characteristics of Registry patients with LOPD who were enrolled in China as of 2 Sep, 2016 and compares these data with findings for patients from the rest of the world.

## Results

### Eligible patients

As of 2 Sep 2016, a total of 1621 patients with Pompe disease had been enrolled to the Registry across 34 countries (Additional file 1: Table S1). In mainland China 78 (4.8%) patients had been enrolled to the Registry at 7 sites located in Beijing, Shanghai, Guangzhou and Jinan, representing the largest number of patients enrolled per country in the Asia Pacific Region. Therefore, the 78 patients enrolled in Mainland China and 1543 patients from the rest of the world (excluding Chinese patients) were eligible for inclusion in this analysis. While China’s contribution represents a small percentage of the total Registry population, patient enrolment in China has increased since the entry of the first patient in 2013. Sufficient information to enable categorization as either IOPD or LOPD had been collected for 64 of the Chinese patients and 1424 patients from the rest of the world, of whom 59 and 1180 had LOPD and were therefore included in the final analysis presented here. Only five Chinese patients with IOPD were entered into the Registry at the cut-off date for this analysis and due to the small patient number these patients were not included in the current analysis.

### Demographic and baseline characteristics

There were several notable differences in characteristics between the Chinese Registry patients and those from the rest of the world. Firstly, among Chinese patients in the Registry the majority had never received primary therapy for Pompe disease (86.4%), in contrast with patients from the rest of the world of whom only 10.4% had never received primary therapy. Chinese patients with LOPD (*n* = 59) had a lower mean age at symptom onset (14.9 [12.35] years) and diagnosis (22.1 [10.08] years) compared with patients from the rest of the world (28.4 [18.86] and 34.9 [20.03] years, respectively) (Table 1). However, the time lapse between onset of symptoms and diagnosis of Pompe disease was similar (~ 5 years) for Chinese patients and patients from the rest of the world. Five (8.5%) of the Chinese LOPD patients had died and were enrolled in the Registry posthumously, and the average age at death was lower compared with patients from the rest of the world; 21.8 (15.81) versus 52.2 (22.07) years.

**Table 1:** Demographics and characteristics of patients with late onset Pompe disease from China versus the rest of the world

Variable, n (%)	China (*n* = 59)	Rest of the world (*n* = 1180)
Male	30 (50.8)	585 (49.6)
Mean age at Pompe symptom onset, years (SD)	14.9 (12.35)	28.4 (18.86)
Mean age at Pompe diagnosis, years (SD)	22.1 (10.08)	34.9 (20.03)
Mean age at first infusion, years (SD)	NA^a^	40.1 (20.02)
Pompe therapy status		
Ever on primary Pompe therapy	< 5	1047 (88.7)
Never on primary Pompe therapy	51 (86.4)	123 (10.4)
Pompe therapy status unknown	7 (11.9)	10 (0.8)
Patients deceased	5 (8.5)	87 (7.4)
Mean age at death, years (SD)	21.8 (15.81)	52.2 (22.07)

*SD* standard deviation. ^a^Data represent one patient and cannot be shown to protect patient anonymity

### Diagnosis of Chinese patients with LOPD

A similar proportion of the Chinese patients with LOPD were diagnosed using either one (49.2%) or more than one (45.8%) method, which was similar to observations for Registry patients from the rest of the world (45.1 and 50.8%, respectively) (Table 2). For Chinese Registry patients, enzyme assay (79.7%) and/or DNA analysis (61.0%) were the most commonly used diagnostic methods, which was also comparable with Registry patients from the rest of the world. Of the 36 patients diagnosed using DNA analysis, 31 had standardized variant data and among these patients the most common mutations were c.2238G > C (*n* = 18, 58.1%) and c.2662G > T (*n* = 5, 16.1%). Interestingly, the most commonly used enzyme assays for patients in China were dry blood spot (46.8%) and ‘other blood-based assays’ (27.7%). In contrast, data from the rest of the world revealed use of a wider range of assays including ‘other blood-based assays’, muscle assays, dry blood spot and fibroblast assays.

**Table 2:** Methods of diagnosis for late onset Pompe disease in China versus the rest of world

Variable, n (%)	China (*n* = 59)	Rest of the world (*n* = 1180)
Number of diagnostic methods used		
One method	29 (49.2)	532 (45.1)
Greater than one method (DNA/enzyme/other)	27 (45.8)	599 (50.8)
Diagnostic methods^a^		
Enzyme assay	47 (79.7)	999 (84.7)
Dry blood spot	22 (46.8)	247 (24.7)
Other blood-based assay	13 (27.7)	455 (45.5)
Fibroblast	< 5	203 (20.3)
Muscle	< 5	314 (31.4)
Unknown/other	< 5	42 (4.2)
DNA analysis, n (%)	36 (61.0)	731 (61.9)
Not reported, n (%)	< 5	49 (4.2)

^a^Patients may report multiple methods of diagnosis, including multiple types of enzyme assays

### Characteristics of Chinese patients with LOPD

**Fig. 1:**
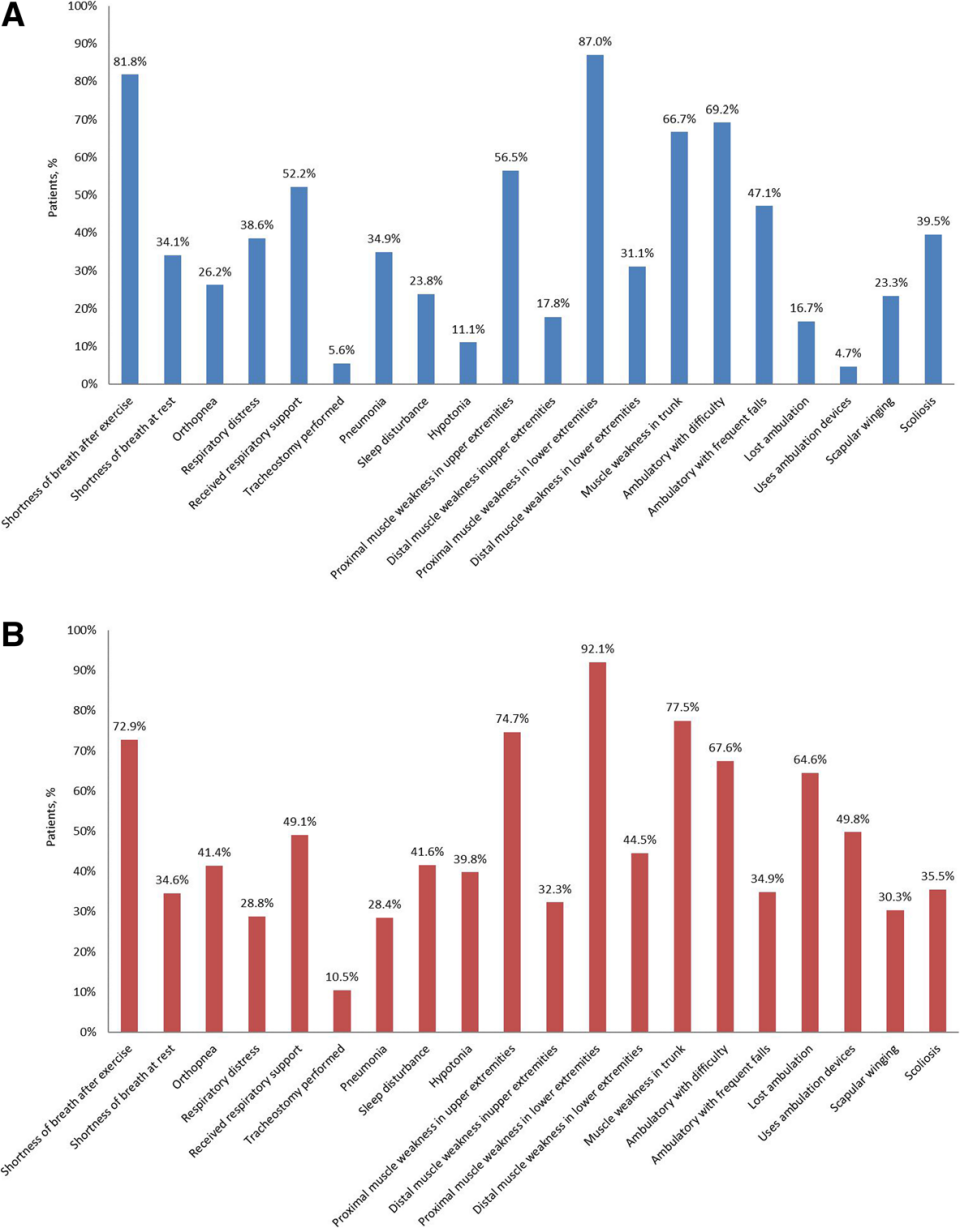
Symptoms of late onset Pompe disease in **a**) China and **b**) rest of the world

The most common (occurring in > 50% of patients) clinical manifestations of Pompe disease for Chinese patients with LOPD were respiratory and musculoskeletal: shortness of breath after exercise (82%), receipt of respiratory support (52%), proximal muscle weakness in lower extremities (87%), ambulatory difficulty (69%), muscle weakness in trunk (67%), and proximal muscle weakness in upper extremities (57%) (Fig. 1). A similar pattern of clinical manifestations was observed among Registry patients from the rest of the world, with the exception of loss of ambulation (65%) and use of ambulation devices (50%) which were not reported for Chinese Registry patients.

### Lung function

In comparison with patients from the rest of the world, PFT results from Chinese patients with LOPD indicated worse respiratory function (Table 3). Of the Chinese patients with LOPD and available PFT data, the percentage with forced vital capacity (FVC) predicted normal, upright and supine, was 37.2 and 29.2%, respectively. In addition, the mean maximal inspiratory pressure (MIP) and maximal expiratory pressure (MEP) were 27.9 cm H_2_O and 29.4 cm H_2_O. All of these values tended to be lower than the average values for Registry patients from the rest of the world.

**Table 3:** Pulmonary function test data for patients with late onset Pompe disease

Variable	China (*n* = 59)	Rest of the world (*n* = 1180)
Patients with ≥1 PFT, n (%)	27 (45.8)	984 (83.4)
FVC % predicted normal (upright) (%)		
n	27	925
Mean (SD)	37.2 (14.00)	63.5 (26.71)
Median (min, max)	35.7 (15.1, 72.3)	62.0 (3.0, 137.0)
FVC % predicted normal (supine) (%)		
n	10	558
Mean (SD)	29.2 (14.41)	55.3 (26.63)
Median (min, max)	23.3 (12.8, 59.4)	53.3 (4.0, 137.0)
MIP (PI_max_) (cm H_2_O)		
n	17	474
Mean (SD)	27.9 (13.54)	51.0 (38.66)
Median (min, max)	24.5 (12.6, 60.9)	45.0 (0.4, 611.8)
MEP (PE_max_) (cm H_2_O)		
n	16	466
Mean (SD)	29.4 (12.04)	70.5 (52.78)
Median (min, max)	26.1 (14.3, 56.7)	62.0 (0.2, 641.4)

*FVC* forced vital capacity, *MEP* maximal expiratory pressure, *MIP* maximal inspiratory pressure, *PFT* pulmonary function test

## Discussion

Due to the low prevalence of Pompe disease, there is often a scarcity of data from large enough populations to allow the characterization of clinical characteristics, phenotype, disease course and treatment outcomes. This is particularly true in China, where previous reports have included relatively small numbers of patients and focused on molecular and genetic characterization of Pompe disease. The Registry provides baseline and longitudinal information on a large global cohort of patients with Pompe disease, and this is the first and largest observational report to focus on the subpopulation of Registry patients in China. Despite this, given that the prevalence of Pompe disease may be as high as 1 in 17,000 [7] and the Chinese population is estimated to be around 1.4 billion, the sample of 59 Chinese patients with LOPD collected in the Registry is small compared to the expected number of cases of Pompe disease in China. This likely reflects the low awareness of Pompe disease among healthcare providers and patients in China, and also the relatively low rate of diagnosis of this condition.

One of the key findings of the present analysis was the lower mean age at symptom onset and diagnosis for Chinese Registry patients with LOPD compared with those from the rest of the world. This finding from the Registry is supported by a previous study of 15 LOPD patients conducted in Taiwan which reported a median age at symptom onset and diagnosis of 15 (range, 10–35) and 21 (10–38) years, respectively [17]. Furthermore, Chinese LOPD Registry patients also had a younger age at death compared with their counterparts from the rest of the world, although due to the small number of Chinese patients who had died, this should be interpreted cautiously. However, it should be noted that the definition of LOPD in this analysis included patients with onset of symptoms at ≤12 months without cardiomyopathy, which differs from previous Registry publications which did not define these patients as LOPD [18]. This may have contributed to the younger mean age at symptom onset and diagnosis.

The mean time delay between Pompe symptom onset and diagnosis observed in the present analysis was similar (~ 5 years) for patients from China and the rest of the world. In comparison, a 2011 Registry report found a delay between symptom onset and diagnosis of around 7 years, and remarked that a reduction in diagnostic delay had been observed since 2004 [18]. Therefore, it is encouraging that the diagnosis gap in the present study is shorter than previous observations, and is similar for Chinese patients and those from the rest of the world. This finding may also reflect the high proportion of Chinese patients diagnosed using the relatively fast blood spot analysis or other blood-based assays. Although, it should be noted that DNA analysis is also an effective method for fast diagnosis. Despite the apparent improvement in time to diagnosis, a delay of several years between symptom onset and diagnosis remains suboptimal considering that disease duration is a key factor for severity of Pompe disease, [19] and that ERT treatment with rhGAA has been shown to slow the progress of disease progression in adults and older children [20]. However, almost none of the Chinese patients in the Registry had received primary ERT treatment for Pompe disease, compared with > 80% of patients from the rest of the world. The main reason for the low rates of ERT treatment observed among Chinese patients in the Registry is that ERT was not available in China until May 2017 and the cutoff for the present analysis was September 2016. It should also be mentioned that the high prevalence of dried blood spot analysis in China may reflect that many Chinese patients with suspected Pompe disease are managed by long distance and send dry blood spots for screening, whereas in Chinese hospitals whole blood is more commonly used for enzyme analyses. In addition, there are only four laboratories in China which can perform the GAA enzyme test which also makes dry blood spot testing more practical for screening. Nevertheless, it is also best practice that diagnoses of Pompe disease are confirmed by a traditional assay, molecular analysis or both.

This analysis found that Chinese Registry patients with LOPD had worse pulmonary function compared with patients from the rest of the world; with lower baseline FVC (upright and supine), MIP and MEP. Furthermore, FVC in the upright position for Registry patients from the rest of the world was 63.5% (26.71), which agrees with earlier reports from the Registry and with previously published studies, and supports the conclusion that Chinese patients have worse pulmonary function [18, 20]. These results suggest that Chinese patients with LOPD have greater diaphragm weakness compared with patients from the rest of the world, which aligns with the higher rates respiratory distress (38.6% vs. 28.8%) reported by these patients compared with their non-Chinese counterparts. Despite the greater impairment of lung function for Chinese Registry patients, the characteristic ≥10% decrease in FVC between upright and supine position was maintained, consistent with the diagnostic criteria for LOPD [21]. However, it should be noted again that the definition of LOPD in this analysis included patients with symptom onset at ≤12 months without cardiomyopathy, and this may have influenced the results compared with studies which defined these patients as atypical IOPD. Our results found that c.2238G > C (p.W746C) was the most common mutation in Chinese Registry patients with LOPD (58.1%), and this is in-line with previous reports in Chinese LOPD patients [16]. In contrast, Caucasian patients with LOPD have a higher prevalence of c.-32–13 T > G (around 40%) [16]. This difference in mutational frequency may explain the worse pulmonary function observed in Chinese patients versus their non-Chinese counterparts. Furthermore, other factors such as living conditions, smoking status of the patient or their immediate family (smoking is more prevalent in China versus many Western countries), and history of lung infections or disease may have influenced these results and further studies are required to clarify this.

It is important to recognize the limitations of the analyses conducted when interpreting the findings presented in this report. The Registry is voluntary and not all patients with Pompe disease have been identified, nor do all wish to participate in the Registry. Therefore, patient numbers for some analyses are very small. Furthermore, although the Registry provides a recommended schedule of assessments, patients and their treating physicians ultimately determine the assessments and the time intervals at which they are carried out. Thus, clinical data may be incomplete for some patients and some assessments or events may be under-reported. Analysis of Registry data will become more meaningful as increased longitudinal clinical data become available for patients in China.

## Conclusions

In conclusion, this first report of Chinese patients in the Registry represents the largest data set currently available on the characteristics of patients with Pompe disease in China. Overall, Chinese Registry patients had a high burden of disease, and the most common mutations were c.2238G > C and c.2662G > T. Compared with patients from the rest of the world, Chinese patients with LOPD appear to be younger at symptom onset and diagnosis, have lower lung function, and are not receiving treatment with ERT. The delay between symptom onset and diagnosis for Chinese patients with LOPD was similar to that in patients from the rest of the world and suggests that even with the common use of blood-based assays there is still room for improvement in the delay between symptom onset and diagnosis.

## Methods

### Registry data collection

The methodology for data collection by the Registry has been described in detail previously [18]. In brief, the Registry is an on-going, international, multi-center, observational program that tracks the routine clinical outcomes for patients with Pompe disease, irrespective of patient age, clinical manifestations or treatment status. All patients with a confirmed diagnosis of Pompe disease, defined as documented GAA enzyme deficiency from blood, skin, or muscle tissue and/or documentation of two GAA gene mutations [3, 22], are eligible for inclusion, and there are no exclusion criteria. However, it should be noted that due to the young age of death associated with IOPD, many patients with this form of Pompe disease are not entered into the Registry. Approval from a local institutional review board/ethics committee was required for inclusion in the Registry. Patient participation is voluntary, and written informed consent is obtained from all participants. The Registry was established in September 2004 and the Registry protocol is registered at ClinicalTrials.gov (NCT00231400).

A recommended schedule of assessments is provided by the Registry [18]. However, the assessments and data collected reflect regional clinical practices, standards of care, and available testing resources. Safety data are not collected by the Registry and healthcare providers are advised to report any spontaneous adverse drug reactions and pregnancy exposures related to ERT directly to the Sanofi Global Pharmacovigilance and Epidemiology Department.

Scientific guidance is provided to the Registry by an independent Registry Board of Advisors comprising physicians and healthcare professionals with extensive scientific and clinical expertise.

### Definitions and measurements

In this analysis, LOPD was defined as presentation after 12 months of age or presentation at ≤12 months without cardiomyopathy.

Diagnostic methods were categorized as: all possible combinations of DNA, enzyme activity, and “other” testing, or as unknown/missing for patients without a reported method of diagnosis or for whom the method was “unknown.” The enzyme diagnostic category includes GAA enzyme activity testing methods that are reported as ‘dried blood spots’, other blood-based (lymphocyte and leukocyte), fibroblast, muscle, or unknown/missing. “Other” includes all write-in responses that could not be categorized as DNA or enzyme analysis, such as muscle biopsy for histologic examination of abnormal glycogen accumulation. More than one type of assay may have been used to diagnose individual patients.

A ‘single’ diagnosis method refers to use of a single enzyme assay method, DNA analysis only, or a single reported “other” method, “more than one” diagnostic method includes any combination of enzyme activity assay, DNA analysis, or other methods, or more than one enzyme method.

### Analyses and statistical methods

All patients with LOPD included in the Registry as of September 2016 were eligible for inclusion in the present analysis. Patient demographics and clinical characteristics are presented using summary statistics. Variables are summarized as mean (SD) unless otherwise stated. Percentages reported for individual measures reflect the proportion of patients with available data for each measure, and are not percentages of the total number of patients included in the analysis. To protect patient anonymity, any result including < 5 patients was summarized as “*n* < 5”. Since all analyses were descriptive, statistical tests were not conducted. All analyses were conducted using SAS 9.2 (SAS Institute Inc., Cary, NC, USA).

## Additional file

Additional file 1: **Table S1.** Enrolment to the Pompe Registry by country as of September 22,016. (DOCX 15 kb)

## Abbreviations

ERT: Enzyme replacement therapy
FVC: Forced vital capacity
GAA: Acid α-glucosidase
IOPD: Infantile onset Pompe disease
LOPD: Late onset Pompe disease
MEP: Maximal expiratory pressure
MIP: Maximal inspiratory pressure
PFT: Pulmonary function test
rhGAA: Recombinant human acid α-glucosidase
