# Supplementary material for: Characteristics of Pompe disease in China: a report from the Pompe registry
Source: Orphanet J Rare Dis. 2019 Apr 3;14:78. doi: 10.1186/s13023-019-1054-0 (PMC6448270; doi:10.1186/s13023-019-1054-0)
Supplement: Supplementary file 1 — Table S1. Enrolment to the Pompe Registry by country as of September 22,016. (DOCX 15 kb) [file 13023_2019_1054_MOESM1_ESM.docx]

Additional Table S1 Enrolment to the Pompe Registry by country as of September 2 2016

| **Patients enrolled per country, n (%)** | **N=1621** |
| --- | --- |
| United States of America | 495 (30.5) |
| Germany | 202 (12.5) |
| Italy | 172 (10.6) |
| France | 156 (9.6) |
| United Kingdom | 145 (8.9) |
| China | 78 (4.8) |
| Netherlands | 72 (4.4) |
| Taiwan | 65 (4.0) |
| Belgium | 40 (2.5) |
| Canada | 35 (2.2) |
| Czech Republic | 22 (1.4) |
| Republic of Korea | 22 (1.4) |
| Israel | 20 (1.2) |
| Poland | 20 (1.2) |
| Brazil | 14 (0.9) |
| Austria | 13 (0.8) |
| Denmark | 6 (0.4) |
| Hong Kong | 6 (0.4) |
| Sweden | 6 (0.4) |
| Hungary | 5 (0.3) |
| Malaysia | * |
| Portugal | * |
| Thailand | * |
| Greece | * |
| Japan | * |
| Chile | * |
| Philippines | * |
| United Arab Emirates | * |
| Australia | * |
| Singapore | * |
| Switzerland | * |

*Country with <5 patients enrolled in the Pompe Registry
